# The Epigenetic Reader Methyl-CpG-Binding Protein 2 (MeCP2) Is an Emerging Oncogene in Cancer Biology

**DOI:** 10.3390/cancers15102683

**Published:** 2023-05-09

**Authors:** Kazem Nejati-Koshki, Chris-Tiann Roberts, Ghader Babaei, Mojgan Rastegar

**Affiliations:** 1 Pharmaceutical Sciences Research Center, Ardabil University of Medical Sciences, Ardabil 85991-56189, Iran; kazemnejati1364@gmail.com; 2 Department of Biochemistry and Medical Genetics, Max Rady College of Medicine, Rady Faculty of Health Sciences, University of Manitoba, Winnipeg, MB R3E 0J9, Canada; rober199@myumanitoba.ca; 3 Department of Clinical Biochemistry, Faculty of Medicine, Urmia University of Medical Sciences, Urmia 57157-89400, Iran; ghaderbiochem@gmail.com

**Keywords:** epigenetics, DNA methylation, MeCP2/MeCP2 isoforms, oncogene, tumor suppressor gene, cancer biology

## Abstract

**Simple Summary:**

Methyl-CpG binding protein 2 (MeCP2) is a nuclear protein that is mainly studied for its role as an epigenetic regulator of gene transcription. Gene mutations affecting the function or expression level of this epigenetic regulator, particularly in the brain, have been associated with a variety of human neurological and neurodevelopmental disorders. However, recent studies suggest that MeCP2, while being an epigenetic reader, also plays a role in the development and/or progression of several types of human cancer. This review provides a general overview of current research into the proposed molecular pathways involving MeCP2 as an emerging oncogene in human cancers and/or cancer cell lines, and highlights potential therapeutic targets in cancer biology.

**Abstract:**

Epigenetic mechanisms are gene regulatory processes that control gene expression and cellular identity. Epigenetic factors include the “writers”, “readers”, and “erasers” of epigenetic modifications such as DNA methylation. Accordingly, the nuclear protein Methyl-CpG-Binding Protein 2 (MeCP2) is a reader of DNA methylation with key roles in cellular identity and function. Research studies have linked altered DNA methylation, deregulation of MeCP2 levels, or *MECP2* gene mutations to different types of human disease. Due to the high expression level of MeCP2 in the brain, many studies have focused on its role in neurological and neurodevelopmental disorders. However, it is becoming increasingly apparent that MeCP2 also participates in the tumorigenesis of different types of human cancer, with potential oncogenic properties. It is well documented that aberrant epigenetic regulation such as altered DNA methylation may lead to cancer and the process of tumorigenesis. However, direct involvement of MeCP2 with that of human cancer was not fully investigated until lately. In recent years, a multitude of research studies from independent groups have explored the molecular mechanisms involving MeCP2 in a vast array of human cancers that focus on the oncogenic characteristics of MeCP2. Here, we provide an overview of the proposed role of MeCP2 as an emerging oncogene in different types of human cancer.

## 1. Introduction

In eukaryotes, the processes of cellular proliferation, differentiation, and function are controlled by genetics and epigenetics. Epigenetics refers to the regulatory mechanisms that control gene expression and distinct characteristics and morphology of the cells without alteration of their corresponding gene sequences [1,2,3,4]. It is well known that genetic mutation of certain genes and/or altered epigenetic mechanisms may cause abnormal cell growth and proliferation. Such deregulation of cellular propagation may trigger the generation of cancerous cells. Indeed, cancer is a heterogeneous disease that is caused by abnormal and uncontrolled cell growth in response to disturbed cellular homeostasis and function. Generally, carcinogenesis is due to the inactivation of apoptotic pathways, activation of oncogenes, and/or inactivation of tumor suppressor genes, resulting in aberrant cell cycle progression and tumor growth [5]. Approximately 1,574,000 persons in Canada have been diagnosed with cancer over a period of 25 years (reported as of 1 January 2018). In particular, breast, prostate, and colorectal cancers accounted for almost half of these 25-year prevalent cancers. In males, prostate and colorectal cancers constituted about half of the prevalent cancers during this time. Among females, breast and colorectal cancers accounted for about half of the prevalent cancers in this report [6]. Overall, these statistics highlight the importance of cancer research towards potential therapeutic strategies.

Cancer initiation and progression may have a genetic and/or epigenetic basis. As such, mutation of certain genes, DNA sequences amplifications, altered DNA methylation, and change in histone post-translational modifications (PTMs) that inhibit tumor suppressor genes or activate oncogenes, are associated with cancer. Furthermore, epigenetic changes can alter cell physiological systems, causing normal cells to transform into tumor cells through deregulated gene activation or inhibition [7,8,9]. As a result, epigenetics are considered as one of the primary contributors to tumor-cell development [10]. Additionally, the growing body of evidence on the role of epigenetics in carcinogenesis has prompted research studies for potential epigenetic drugs for tumor cells.

## 2. Interplay between DNA Methylation and Cancer

There are different forms of epigenetic modifications that include chemical changes in DNA molecules and histone PTMs with regulatory roles in gene expression [11,12]. DNA methylation is involved in biological processes ranging from regulation of gene expression, RNA splicing, genome stability, X chromosome inactivation, cellular metabolism, and imprinting [13,14]. DNA methylation refers to the covalent modification of DNA molecules at the fifth position of the cytosine pyrimidine ring, producing 5-methylcytosine (5mC, 5-methyly C, or C^Me^). This process is mediated by the action of DNA methylation “writers”, a family of enzymes known as DNA methyltransferases (DNMTs) [15,16]. Specifically, DNA methyltransferases transfer a methyl group donated by S-adenosyl-L-methionine (SAM) to the cytosine residue of CpG dinucleotides [17]. In addition to 5mC, there is another methylation in the structure of DNA, such as N6-adenine methylation (6mA), which has recently been reported in *Caenorhabditis elegans* and mammals and is frequently associated with gene transcriptional activation [18].

DNA methyltransferases are classified into three classes: DNMT1, DNMT2, and DNMT3A/3B/3L [19]. DNMT1 is the maintenance DNA methyltransferase and preserves the methylation pattern in the replication fork during DNA synthesis. Meanwhile, DNMT3A/3B (de novo DNA methyltransferases) participate in de novo DNA methylation during development and gametogenesis [19,20]. In contrast, DNMT2 (also known as TRDMT1) does not act as a DNA methyltransferase, yet its function is related to positioning methylation in aspartic acid-transfer RNA [19,20]. DNMT3L is reported to be unable to methylate DNA molecules. The amino acid sequence of DNMT3L bears similarity to DNMT3A and DNMT3B; however, it lacks the required C-terminal amino acid residues that are responsible for DNMT enzymatic activities [21].

Based on the “G” and “C” contents of the human genome, CpG dinucleotides are not as frequent throughout the genome as expected. On the other hand, CpG islands have a higher frequency of CpG dinucleotides [22]. Almost half of human gene promoters contain CpG islands [23]. Methylation of these CpG-rich sequences is involved in transcriptional gene regulation. In mammals, promoter DNA methylation is typically linked to the suppression of gene expression, while demethylation in the gene regions is believed to activate gene expression. However, depending on the type of DNA methylation (i.e., 5mC versus 5-hydroxymethyl cytosine (5hmC)), the regulatory mechanism might be different. There are different scenarios on how DNA methylation controls gene expression. It is possible that methylated DNA directly recruits transcription factors that inhibit transcription such as transcriptional inhibitors. Alternatively, methylated DNA may attract chromatin remodeling complexes and recruit methyl-binding proteins (MBPs) [15]. Methylation of CpG dinucleotides creates binding sites for methyl-CpG-binding domain (MBD) proteins such as MBD1, MBD2, MBD3, MBD4, MeCP2, and Kaiso, which control gene expression through protein–protein interactions and the recruitment of chromatin remodeling factors [16,24]. Among MBDs, MeCP2 is known for its role in the organization of the chromatin structure, chromatin-loop formation, recruitment of corepressors (acting as a transcriptional inhibitor), or activators (acting as a transcriptional activator) [11,25,26]. Interestingly, the murine *Mecp2*/MeCP2 is also influenced by DNA methylation [27,28,29,30,31]. Furthermore, MeCP2 plays regulatory roles in cellular metabolism, the mTOR pathway, and the control of protein translation initiation which are important in cellular proliferation [14,32,33,34]. Independent research has suggested that MeCP2 is involved in cancer [35,36], a subject that we will discuss here for different types of human cancer.

One characteristic of cancer cells is the extensive change in their DNA methylation patterns. Typically, global change in DNA methylation happens during early development with substantial changes during stem-cell differentiation [12,37,38,39,40]. In differentiated cells, however, minimal change in DNA methylation occurs, which could still be influenced by environmental factors. In fact, DNA methylation of differentiated cells is maintained during DNA replication due to the high affinity of DNMTs for hemi-methylated DNA [41]. Interestingly, global change in the DNA methylation of cancer cells was among the first epigenetic aberrations that were noted in human cancers [42]. The *RB1* gene was the first tumor-suppressor gene reported to be inhibited through DNA hypermethylation [43]. This prompted researchers to study CpG islands as the promoter of tumor suppressor genes. Evidence shows that DNA hypomethylation may relate to chromosome instability, while DNA hypermethylation of tumor-suppressor genes inhibits their expression [44].

A primary piece of evidence on the role of aberrant DNA methylation in tumorigenesis was presented by Herman and colleagues in 1994. They showed that hypermethylation of the tumor suppressor gene *VHL* may lead to its transcriptional silencing in renal carcinomas. Since then, extensive studies have shown that hypermethylation of the promoter region of tumor suppressor genes is involved in their transcriptional silencing in human cancers. Cancer-related DNA methylation is often considered as a primary step for neoplastic transformation. This suggests that DNA methylation may act as an initial link between the impact of the environment on cancer. This may be interpreted as a link between lifestyle and cancer-related DNA methylation in initially asymptomatic individuals. Thus, DNA methylation is assumed to be one of the several initiators of many human cancers [45]. In addition to hypermethylation of promoters and other *cis*-regulatory elements of tumor suppressor genes, there are other mechanisms such as reduced DNA methylation in the cis-regulatory regions of oncogenes, inducing cellular proliferation and cancer. Of note, chromosome instability, which results from genome-wide DNA hypomethylation, is also involved in cancer development and tumorigenesis [45,46].

### MeCP2 and DNA Methylation

MeCP2 was the first discovered member of the MBD family of proteins reported in 1992 by Adrian Bird and colleagues, when they aimed to study the factors that prevent CpG islands from DNA methylation [47,48]. Different MeCP2 protein domains include the N-terminal domain (NTD), methyl-CpG-binding domain (MBD), intervening domain (ID), transcription repression domain (TRD) with nuclear localization sequence (NLS), NCoR interaction domain (NID), and C-terminal domain (CTD) [49,50,51]. *MECP2* is an X-linked gene located on the Xq28 chromosome. Both the murine (*Mecp2*) and human (*MECP2*) genes contain four exons, encoding for the two well-studied protein isoforms produced by alternative splicing, namely MeCP2E1 (also identified as MeCP2B or MeCP2α) and MeCP2E2 (also recognized as MeCP2A or MeCP2β). MeCP2E1 and MeCP2E2 isoforms only differ in their N-termini, with additionally predicted coding and noncoding *MECP2* isoforms [52]. MeCP2E1 includes exons 1, 3, and 4, and it is the prevalent isoform in the brain [29,53,54]. MeCP2E2 contains exons 2, 3, and 4, and is slightly shorter than MeCP2E1. MeCP2E2 has 486 amino acids with an expected molecular weight of 53 kDa, although it is commonly detected around 72–75 kDa [27,29,32,47,48,54]. Recently, the regulation of *MECP2* isoforms by metabolic drugs (metformin and simvastatin) was studied in a medulloblastoma brain-tumor cell line [55]. Interestingly, simvastatin causes cell death and apoptosis in different subgroups of medulloblastoma brain tumors [56]. In addition to the MeCP2 function in transcriptional repression, the protein is also capable of transcriptional activation [26]. Such diversity of MeCP2 function may result from its wide range of interaction protein partners; some may be specific to one particular protein isoform [57].

## 3. An Overview of the Genetics of Cancer: Tumor Suppressor Genes and Oncogenes

At large, the genetic basis of cancer initiation and progression may implicate two distinct groups of genes with opposite functions, known as tumor-suppressor genes and oncogenes. In general, tumor-suppressor genes encode for protein factors that could act as negative regulators of cell survival and proliferation [58]. In contrast, oncogenes encode for protein factors that may be crucial for normal cell function [59]. Years after their original discovery, both groups are proven to be determinants of tumor classification, prognosis, response to cancer-related therapies, and indicators of clinical outcomes [60,61,62,63,64]. 

### 3.1. A Brief History 

Fifty years following Theodor Boveri’s hypothesis of numerical change in chromosomal content involved in tumorigenesis, a chromosomal abnormality was reported in patients with chronic myelogenous leukemia [65,66]. During the 1970s, Alfred Knudson hypothesized that there is a specific class of cancer-related genes that may act recessively and require “two hits” for tumorigenesis [67]. This hypothesis led to the cloning of the first tumor-suppressor gene [67,68,69]. By the late 1970s, the first proto-oncogene, *c-scr*, was identified [70]. In the following years, the first oncogenic activation of *H-RAS* was described [71]. Since then, an arsenal of molecular and cellular biology techniques ranging from comparative genomic hybridization, functional analysis, site-directed mutagenesis, and advanced genetic techniques, including the application of viral vectors, have been employed in the identification of amplified/overexpressed or mutated cancer-related genes, possible therapeutic strategies, and mechanisms of drugs’ actions in different types of human cancer [72,73,74,75,76].

### 3.2. Tumor-Suppressor Genes and Oncogenes: Mechanism of Action

Tumor-suppressor genes and oncogenes may both undergo genetic mutations, but they are functionally distinct regarding cancer initiation and/or progression. Tumor-suppressor genes are recessive and may carry biallelic loss-of-function mutations to promote cancer malignancy [77]. In this case, the transformation of normal cells to cancerous cells could be associated with the elimination of one or more tumor suppressor genes. Typically, there is a mutation in one allele of a tumor suppressor gene along with deletion in the other allele to constitute a total loss of function for the tumor suppressor gene leading to the manifestation of malignant phenotypes [67]. Alternatively, other mechanisms such as methylation of the gene promoter may inhibit their expression [59]. Amongst the best-characterized tumor suppressor genes are *p53*, *p27*, *CHK2*, *ATM*, and *RB,* with key roles in the eukaryotic cell cycle. Additionally, *BRCA1* and *BRCA2* tumor suppressor genes contribute to cellular function such as DNA damage repair [78]. On the other hand, gain-of-function mutation of proto-oncogenes may lead to an elevated level of oncogenes that could act dominantly with their single-copy mutation, being sufficient to promote carcinogenesis [79]. Oncogene activation may happen through several different mechanisms including (i) chromosomal translocation, (ii) gene amplification, and (iii) point mutation. Overall, oncogene activation could also involve changes in proto-oncogene structure or increased expression of proto-oncogenes. As such, chromosomal rearrangements, i.e., chromosomal translocations associated with carcinogenesis, may occur via the formation of oncogenic fusion proteins or activation of an oncogene in response to a new promotor element or enhancer sequence [80]. Moreover, increased gene copies, or gene amplification, can occur via redundant DNA replication [81]. Furthermore, point mutations (alteration, insertion, or deletion of one or more nucleotides) may potentially activate oncogenes by enhancing the function of the oncoprotein [59]. Of note, *HER2* gene amplification, *RAS* point mutations, and *MYC* chromosomal translocation are associated with tumorigenesis in breast cancer, thyroid cancer, and Burkitt’s lymphoma, respectively [82,83,84].

## 4. Connections between MeCP2 and Human Cancer

The involvement of MeCP2 in Rett Syndrome (RTT), a neurological disorder in females, has been well-documented [85,86,87,88,89]. Over 90% of RTT patients possess a mutation in the *MECP2* gene [87]. However, in recent years, many studies have linked MeCP2 to different types of human cancer. It is reported that MeCP2 is overexpressed in many human cancers, and, when knocked down, leads to reduced propagation of cancer cells [90,91]. Additionally, studies on the relationship between MeCP2 and clinicopathological parameters of cancers have suggested that MeCP2 expression levels in cancer are often high and are effective in regulating the development of tumors [92,93,94,95,96,97]. Unbiased genome-scale screenings have indicated that the two alternatively spliced isoforms of *MECP2* when overexpressed/amplified, activate the MAPK and PI3K/RAS-induced growth factor pathways as MeCP2 acts as a substitution for activated RAS [90]. Fitting with the emerging role of MeCP2 in the pathology of different types of human cancer in recent years, extensive research has been conducted to better understand the mechanisms by which MeCP2 acts as an oncogene in different types of cancer. In the following sections, we will discuss the reported evidence on how MeCP2 contributes to the development of various types of human cancer.

### 4.1. MeCP2 and Breast Cancer

**Figure 1:**
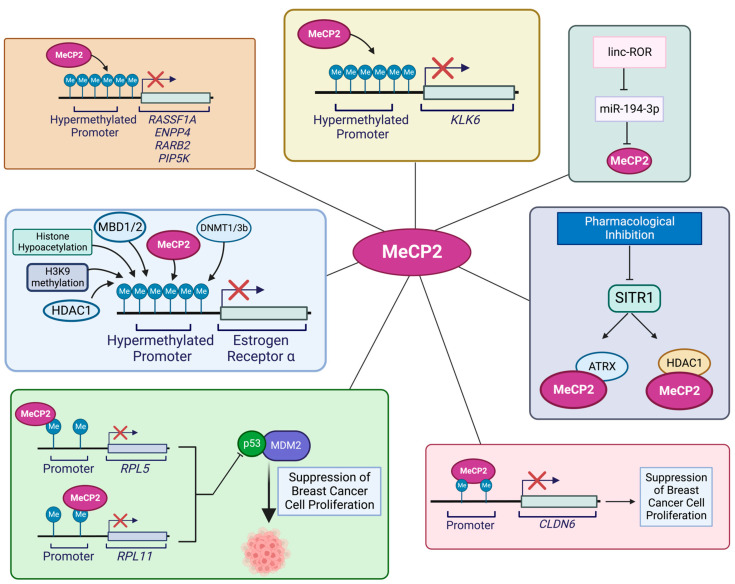
Schematic representation of suspected roles of and molecular pathways involving MeCP2 in breast cancer. DNA hypermethylation, histone hypoacetylation, H3K9 methylation, and binding of MeCP2, and other proteins shown in this Figure that will contribute to ER silencing [103]. Binding of MeCP2 to the hypermethylated of CpG sites in the proximal region of the *KLK6* promoter silences the *KLK6* gene [105]. Binding of MeCP2 to the promoter region of *RPLL1* and *RPL5* reduces the expression levels of their respective proteins and disrupts their inhibitory activity in ubiquitination-mediated p53 degradation alongside the binding to MDM2 [35]. MeCP2 acetylation levels are regulated by the interaction between SIRT1, ATRX, and HDAC1 in breast cancer [106]. Recruitment of MeCP2 to the methylated regions of *CLDN6*, along with deacetylation of histones H3 and H4, results in the invasion and migration capacity of breast-cancer cells [107]. MeCP2 inhibition by *miR-194-3p* may be achieved via binding to the *MECP2* 3′-UTR. However, linc-ROR acts as a competitive endogenous RNA for *miR-194-3p* [108]. MeCP2, in relation to MeCP2-mediated regulation of tumor-suppressor genes, is found in association with the *PIP5K*, *RASSF1A*, *ENPP4*, and *RARB2* gene promoters [109]. Illustration is generated using BioRender.com.

Breast cancer has the highest frequency of diagnosis and constitutes about 25% of all cancer cases in females (based on Global Cancer Statistics 2020) [98,99]. Studies on the role of epigenetics in the progression of breast cancer have established a link between MeCP2 and breast cancer [100]. Specifically, a high MeCP2 expression level in breast cancer is associated with its recruitment to hypermethylated sequences at the regulatory regions of tumor-suppressor genes [35]. Some of the molecular pathways involving MeCP2 in breast-cancer progression are summarized in Figure 1. In a previous study, Billard and colleagues [101] showed that both MBD2 and MeCP2 expression levels are linked to the proliferation of normal breast tissue as well as benign and neoplastic breast tumors. The authors reported that MBD2 and MeCP2 expression levels were different in breast cancer versus normal samples, indicating their possible role in breast cancer [101]. In another study on breast cancer tissue specimens, Müller and colleagues reported an association between MeCP2 and estrogen receptor (ER), with higher MeCP2 levels in estrogen receptor (OR)-positive breast cancer samples compared to OR-negative ones [96]. In another study, Sharma and colleagues explored the role of MeCP2 in modulating ER transcriptional silencing in OR-negative breast cancer cells. Sharma and colleagues further reported that increased DNA methylation, reduced histone acetylation, along with H3K9 methylation, MeCP2 binding, and recruitment of other factors such as MBD1, MBD2, DNMT1, DNMT3B, and HDAC1 lead to estrogen receptor silencing. Furthermore, the authors demonstrated that DNMT inhibition with 5-aza-2′-deoxycytidine (5-aza) and HDAC inhibitor Trichostatin A (TSA) may result in ER re-expression. Treatment of MDA-MB-231 breast cancer cells with TSA or 5-aza leads to re-expression of ER. In such a case, there was a synergistic effect when treating these cells with a combination of both 5-aza and TSA. This led to the dissociation of a repressor complex containing MeCP2, DNMTs, and HDAC1 from the ER promoter, resulting in ER re-expression. The authors reported similar results in MDA-MB-468 cells, showing that in these cells DNMT1, HDAC1, and MeCP2 silence estrogen receptor 1 (ESR1) [102]. 

There are different mechanisms of MeCP2 involvement in breast cancer [103]. Jiang and colleagues reported that in breast-cancer cells, MeCP2 can inhibit the epithelial-to-mesenchymal transition (EMT) and their migration. The authors demonstrated that MeCP2 overexpression induces epithelial markers, while MeCP2 knockdown increases mesenchymal markers [104]. The tumor suppressor kallikrein-related peptidase 6 (KLK6), is inactivated in metastatic breast-cancer cells, and studies have underscored the role of MeCP2 in silencing the KLK6 gene in metastatic breast cancer. It is suggested that the tumor-specific loss of KLK6 expression is due to increased DNA methylation at the KLK6 promoter. This was associated with MeCP2 binding to the hypermethylated *KLK6* promoter, histone deacetylation, and repressive chromatin formation that silenced *KLK6* [105].

There are reports of other types of mechanisms for MeCP2 involvement in breast cancer. In this scenario, MeCP2 was found to bind to promoters of the ribosomal protein L11 (RPL11) and the ribosomal protein L5 (RPL5) genes, reducing the expression level of their corresponding proteins. Typically, expression of RPL11 and RPL5 leads to suppressed breast-cancer cell proliferation via inhibition of ubiquitination-mediated p53 degradation and inhibition of its binding to MDM2. The researchers observed that MeCP2 induced breast-cancer cell proliferation while reducing apoptosis via ubiquitination-mediated P53 degradation by suppressing *RPL5* and *RPL11* expression [35]. Pandey and colleagues studied the role of SIRT1 in regulating MeCP2 function. They showed that SIRT1 is involved in MeCP2 interaction with ATRX and HDAC1 through regulation by controlling MeCP2 acetylation levels. Among the lysine residues in MeCP2, lysine 171 and its acetylation are involved in MeCP2 protein interactions with ATRX and HDAC1. In support of this mechanism, studies in MeCP2-K171Q mutant cells have shown a significant decrease in its binding to ATRX and HDAC1 as compared to cells with wild-type MeCP2. This indicates a role for MeCP2 PTMs in the regulation of its function [106]. Liu and colleagues suggested another mechanism of the MeCP2 role in breast-cancer metastasis involving claudin-6 (CLDN6), a member of the claudin transmembrane protein family. CLDN6 inhibits breast-cancer metastasis and cell invasion and has low expression levels in breast cancer. Studies have shown that CLDN6 gene methylation is associated with MeCP2 binding, along with histone H3 and H4 deacetylation, leading to reduced CLDN6 expression, resulting in increased invasion and breast-cell migration [107]. 

Zhou and colleagues proposed another mechanism of action for MeCP2 in breast cancer, involving the microRNA *miR-194-3p*. They showed that *miR-194-3p* inhibits MeCP2 expression by binding to the 3′-UTR of the *MECP2* gene. However, in breast cancer, linc-ROR, which has a high expression level in breast cancer tissues and plasma, can act as a competitive endogenous RNA (ceRNA) for *miR-194-3p*. This interaction hinders the inhibitory effect of this microRNA on *MECP2* once bound to *miR-194-3p*, and, instead, increases cell proliferation, migration, and invasion, while decreasing cellular drug sensitivity to rapamycin [108].

### 4.2. MeCP2 and Colorectal Cancer

**Figure 2:**
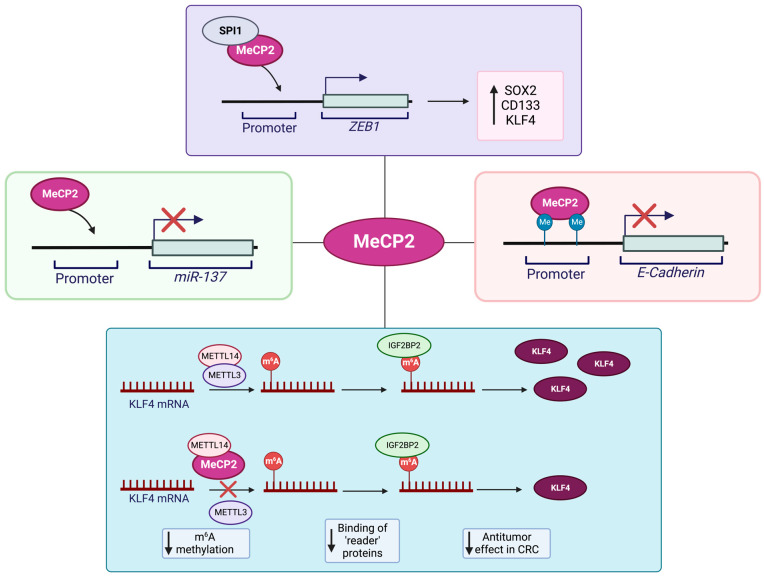
Schematic representing the suspected roles of and molecular pathways involving MeCP2 in colorectal-cancer progression. MeCP2 leads SPI1 to the *ZEB1* promoter to increase ZEB1 expression. Increased ZEB1 expression leads to upregulation of MMP14, SOX2, and CD133 [91]. MeCP2-mediated regulation of KLF4 is achieved through the binding of MeCP2 to METTL14. Complexing of MeCP2 and METTL14 results in decreased m6A methylation, and destabilization of KLF4 transcripts and CRC metastasis [111]. MeCP2, in conjunction with DNA methylation at the promoter region, regulated the expression of E-cadherin in CRC [112]. MeCP2-mediated epigenetic silencing of *miR-137* is also shown [113]. Illustration is generated using BioRender.com.

Research studies have proposed a role for MeCP2 in the pathogenesis of colorectal cancer (CRC). Given the shared role of the MeCP2 mutations in causing the neurological disorder RTT and its association with colon cancer, there are some concerns in patients with RTT and their risk of developing colon cancer [110]. The MeCP2 level is high in adenocarcinoma and mucinous adenocarcinoma tissues, specifically at the colorectal cancer invasion sites. Song and colleagues reported that DLD-1 cells had high MeCP2 expression levels in different colorectal cancer cell lines. The authors showed that lentivirus-mediated knockdown of MeCP2 inhibits the G0/G1 cell-cycle progression, inhibiting the migration ability of these cells [97]. Luo and colleagues studied the relation of MeCP2 with that of CRC stemness and metastasis. They reported that in colorectal cancer, MeCP2 has a high expression level that is correlated with poor patient prognosis. On the other hand, MeCP2 depletion (KO/KD) inhibited invasion and migration (in vitro) as well as metastasis (in vivo). The research group also showed that MeCP2 interacts with SPI1, being recruited at the *ZEB1* promoter, resulting in increased expression of the corresponding protein. Following elevated ZEB1 expression, they detected induced MMP14, CD133, and SOX2 and stemness maintenance that promoted CRC metastasis [91]. Wang and colleagues proposed another mechanism to link MeCP2 with CRC metastasis. This group showed that MeCP2 regulates Kruppel-like factor 4 (KLF4) expression through binding to methyltransferase-like 14 (*METTL14*) and changing RNA N6-methyladenosine (m6A) methylation. More detailed studies showed that the interaction of these two proteins decreases m6A methylation. Given that KLF4 stability as a tumor suppressor depends on the interaction of m6A methylation reader protein IGF2BP2 with two unique m6A methylation sites in the *KLF4* transcript. This leads to reduced m6A methylation that causes *KLF4* transcript instability, reduced expression level, and, eventually, induces CRC metastasis [111]. Darwanto and team further reported that E-cadherin expression in colorectal cancer is regulated by promoter methylation and linked to MeCP2 expression [112]. Additionally, Chen and colleagues reported that *miR-137* is one of the inhibitors of CRC development which is affected by epigenetic regulation mediated by MeCP2 and is associated with *miR-137* silencing [113]. Different molecular mechanisms of MeCP2 involvement in CRC progression are summarized in Figure 2.

### 4.3. MeCP2 and Pancreatic Cancer

**Figure 3:**
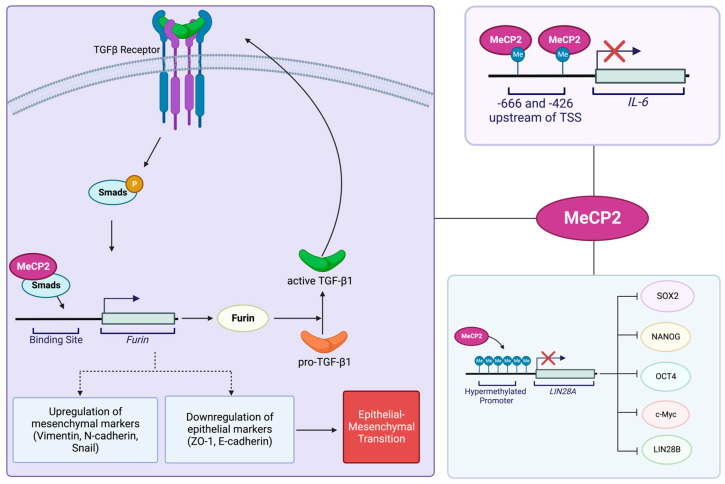
Schematic representing the suggested roles of and molecular pathways involving MeCP2 in pancreatic cancer. MeCP2 increases mesenchymal markers including snail, N-cadherin, and vimentin. MeCP2 simultaneously decreases epithelial markers including E-cadherin and ZO-1 to induce EMT. The increase and decrease of mesenchymal and epithelial markers, respectively, are achieved through the binding of MeCP2 to the *Furin* promoter and subsequent activation of TGF-β1 by Furin and Smad phosphorylation [115]. MeCP2 recruitment to the methylated CpG islands of *LIN28A* hinders the ability of LIN28A to increase the expression of NANOG, c-Myc, OCT4, SOX2, and LIN28B [116]. MeCP2 binding to methylated CpG sites at the *IL-6* gene may result in its silencing [117]. Illustration is generated using BioRender.com.

The association of MeCP2 with pancreatic-cancer pathogenesis has also been investigated. Independent groups have studied the underlying molecular mechanisms of MeCP2 involvement in pancreatic-cancer progression, which we have summarized in Figure 3. Xie and colleagues reported that in patients with pancreatic cancer, the MeCP2-positive expression rate is lower in cancer tissues compared to the adjacent normal cells. Their studies also indicated that patients who are positive for MeCP2 have longer survival compared to those patients that do not express MeCP2. These findings imply that MeCP2 detection may be beneficial in the prognosis of pancreatic-cancer patients [114]. Wang and colleagues reported a link between MeCP2 and EMT in pancreatic ductal adenocarcinoma (PDAC). MeCP2, which is highly expressed in pancreatic cancer, was shown to increase mesenchymal markers that include vimentin, snail, and N-cadherin. Contrastingly, MeCP2 expression decreased specific epithelial markers such as E-cadherin and ZO-1 and induced EMT. These changes are suggested to be the result of a positive-feedback regulatory network, where MeCP2 binds to the *Furin* promoter through Smad3 interaction, increasing its expression. Here, Furin acts as an activator of TGF-β1, inducing Smad2/3 phosphorylation [115].

Xu and colleagues proposed that LIN28A, an effective factor in cancer malignancies, has a high level of expression in PANC1 cells. LIN28A enhances the characteristics of stem cells in pancreatic-cancer cells by inducing SOX2, OCT4, LIN28B, c-Myc, and NANOG. The authors showed that MeCP2 acts as a regulator of LIN28A and binds to methylated-CpG islands to inhibit LIN28A expression in pancreatic-cancer cells [116]. Other studies involved MeCP2 in the repression of interleukin-6 (IL-6), a key cytokine involved in a variety of biological activities in pancreatic cancer. However, the mechanism of IL-6 repression by MeCP2 has yet to be fully explored. In this regard, Dandrea and colleagues showed that MeCP2 may bind to methylated CpG located from positions −666 to −426 of *IL-6* and be associated with H3meK9 at this locus. In this way, both MeCP2 and H3meK9 are involved in the silencing of *IL-6* in pancreatic-adenocarcinoma cell lines [117].

### 4.4. MeCP2 and Gastric Cancer

**Figure 4:**
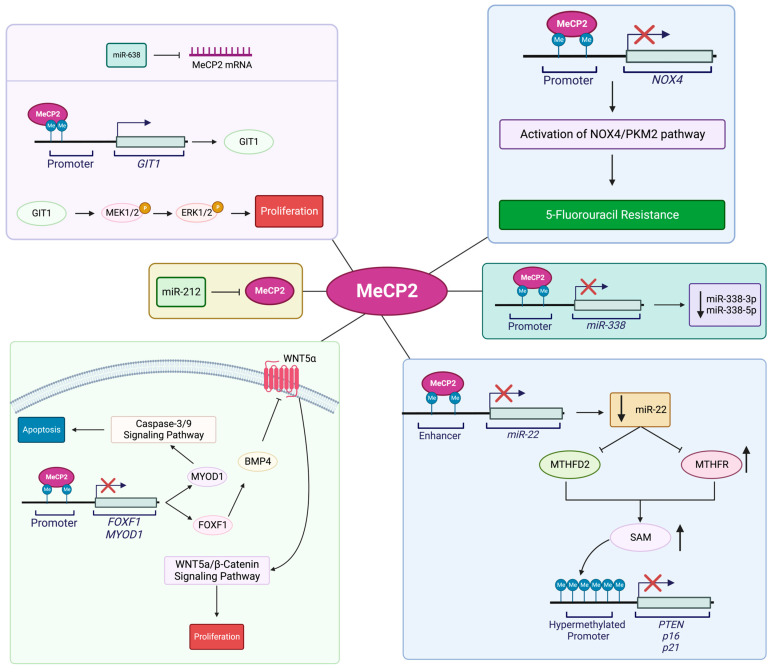
Schematic representing the suggested roles of and molecular pathways that link MeCP2 to gastric-cancer progression. MeCP2-mediated activation of Wnt5a/β-Catenin signaling via FOXF1 inhibition promotes cell proliferation, while MeCP2-mediated inhibition of the caspase-3 signaling via MYOD1inhibition promotes apoptosis [93]. Inhibition of MeCP2 may be achieved by *miR-638*. Binding of MeCP2 to the promoter region of *GIT1* increases GIT1 expression. GIT1 activates the MEK1/2-ERK1/2 signaling pathway and promoted cell proliferation [120]. Expression levels of MeCP2 are reduced by the binding of *miR-212* to the *MECP2* 3′UTR region [121]. Upregulation of MeCP2 results in increased expression of NOX4 via binding of MeCP2 to the *NOX4* promoter region and upregulation of the NOX4/PKM2 pathway in 5-FU resistance [123]. Inhibition of MTHFD2 and MTHFR, and increased level of PTEN, p16, p21, and RASSF1A, by the action of *miR-22*, results in decreased cell proliferation. However, MeCP2-mediated inhibition of *miR-22* deregulates tumor suppression through the action of *miR-22* in gastric-cancer cells [124]. Gastric-cancer cell proliferation is due to MeCP2-mediated inhibition of *miR-338-3p* and *miR-338-5p* [125]. Illustration is generated using BioRender.com.

It has been shown that MeCP2 deregulation impacts physiological processes related to the development of gastric cancer (GC), as summarized in Figure 4. Examining tissues from patients with diagnosed gastric cancer has shown that MeCP2 expression is significantly higher in GC samples compared to para-cancerous tissues. MeCP2 expression levels were significantly correlated with tumor, node, and metastasis (TNM) stages, histological types, and status of lymph node metastasis. These results indicated that MeCP2 may regulate GC development [92]. Zhao and colleagues reported that MeCP2 binds to methylated CpG islands of *FOXF1* and *MYOD1* promoters, inhibiting the protein levels. Studies have shown that MeCP2 activates Wnt5a/β-Catenin signaling, promotes GC cell growth, promotes G1/S cell-cycle progression via FOXF1 inhibition, inhibition of caspase-3 signaling, and apoptosis by MYOD1 inhibition [93]. In another study, researchers found that MeCP2 inhibited FBXW7 expression in GC cells by binding to methylated CpG sites in the *FBXW7* promoter, playing important roles in blocking cell migration and invasion. Reduced FBXW7 expression activated the Notch1/c-Myc/mTOR signaling pathways, and promoted GC cells migration and invasion [118].

Another study explored the relation between MeCP2 and human cancer regarding the role of microRNAs in controlling MeCP2. In this context, scientists have shown that disrupting the regulatory role of microRNA on MeCP2 is involved in different types of human cancer. For instance, Zhang and colleagues showed that the expression of microRNA-1324 in GC decreased when MeCP2 expression was high. The authors showed that MeCP2 may be a target of microRNA-1324 by inhibiting *MECP2* expression by direct binding. More detailed studies showed that reduced microRNA-1324 expression has the opposite function with GC proliferation and invasiveness, in association with MeCP2 inhibition [119]. Zhao and co-authors also reported that *miR-638* is involved in the regulation of MeCP2 in GC. This team of researchers showed that *miR-638* repressed GC cell division, colony creation, G1/S cell-cycle transition, and tumor progression by binding to *MECP2* 3′-UTR, leading to induced apoptosis. Further studies showed that MeCP2 binds to the promoter of G-protein-coupled receptor kinase-interacting protein 1 (*GIT1*), increases GIT1 protein expression, and activates the MEK1/2-ERK1/2 signaling pathway to promote gastric-cancer cell proliferation [120]. It is also shown that MeCP2 acts as a target of *miR-212*. Wada and colleagues reported that *miR-212* can reduce MeCP2 expression levels by binding to the *MECP2* 3′-UTR region. However, *miR-212* expression has decreased in GC cell lines and primary GCs, resulting in an increased MeCP2 level that is correlated with GC carcinogenesis [121]. Other studies described the relation of MeCP2 with multidrug resistance (MDR). These studies suggested that demethylation of *miR-19a/b*, which is highly expressed in GC, is also involved in the MDR of gastric cancer via binding to the *MECP2* 3′-UTR. Such interactions inhibited *MECP2* expression, reducing the inhibitory effect of MeCP2 on *miR-19a/b* and, eventually, inducing MDR in gastric cancer. These results also indicated that there is an opposite relationship between *miR-19a/b* expression with that of MeCP2 in gastric cancer [122]. Qin and co-authors studied MeCP2 involvement in 5-FU-resistant GC cells, reporting that MeCP2, which is upregulated in 5-FU-resistant GC cells, increases NOX4 by binding to the *NOX4* promoter sequences. NOX4 is a highly expressed NOX isoform in cancer cells, mediating drug resistance via PKM2. By upregulating the NOX4/PKM2 pathway, MeCP2 contributes to 5-FU resistance in GC cells [123]. Furthermore, Tong and colleagues studied the link between MeCP2 and *miR-22* in GC. This microRNA inhibits MTHFD2 and MTHFR, both of which are effective enzymes in the synthesis of SAM. This microRNA is also involved in reducing DNA methylation and increasing the expression of PTEN, p16, p21, and RASSF1A in the inhibition of cellular proliferation. The results of this group showed that in GC, MeCP2 inhibits *miR-22* expression in GC cells by binding to its upstream methylated enhancer, causing tumor suppressor deregulation [124]. Tong and colleagues also reported that MeCP2 binds to the methylated-CpG island of the *miR-338* promoter, inhibiting *miR-338-3p* and *miR-338-5p*, and promoting GC cell proliferation [125].

### 4.5. MeCP2 and Hepatocellular Carcinoma (HCC)

**Figure 5:**
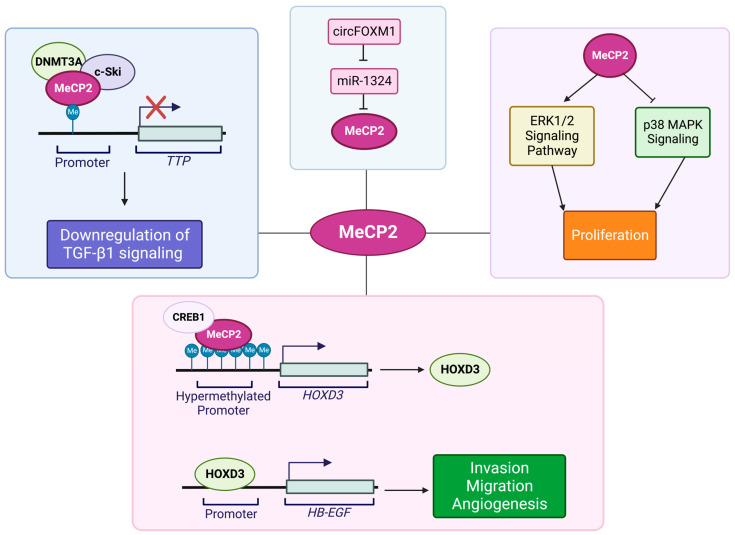
Schematic representing the suspected roles of and molecular pathways involving MeCP2 in hepatocellular carcinoma (HCC). Hypermethylation of the *TTP* promoter, and subsequent repression of TTP expression, is involved in the downregulation of TGF-β1 signaling in the progression of HCC via the recruitment of HDAC complexes by MeCP2 [126]. Activation of the ERK1/2 signaling pathway and inhibition of p38 activity by MeCP2 promotes cell proliferation in HCC [127]. Binding of MeCP2 and CREB1 to the hypermethylated *HOXD3* promoter increases the expression of HOXD3. Binding of HOXD3 to the *HB-EGF* promoter results in increased HB-EGF activation and increased cell invasion and migration of HCC cells [129]. Negative regulation of MeCP2 in sorafenib resistance by *miR-1324* is alleviated in the presence of circFOXM1, leading to increased expression levels of MeCP2 [130]. Illustration is generated using BioRender.com.

Research studies in hepatocellular carcinoma cell lines and patient tissues have suggested another role for MeCP2 in cancer. Our current knowledge about the molecular pathways that link MeCP2 and hepatocellular carcinoma (HCC) is summarized in Figure 5. In a study about transforming growth factor (TGF) β1 (TGF-β1) signaling in liver cancer, Sohn and colleagues proposed that tristetraprolin (TTP) promoter hypermethylation contributes to TGF-β1 deregulation in the malignant progression of hepatocellular carcinoma [126]. TTP is suggested to act as a tumor suppressor, while TGF-β1 regulates cell growth and differentiation, as well as the migration and apoptosis of hematopoietic and epithelial cells. In cancer biology, TGF-β1 is considered to both promote tumor-growth progression and suppression that are associated with deregulated TGF-β1 signaling. As such, deregulated TGF-β1 signaling permits cancer cell angiogenesis, invasion, and metastasis. The deregulated TGF-β1 pathway in hepatocellular carcinoma cells involves methylation of one out of 90 CpG sites in the *TTP* promoter. De novo methylation of a CpG dinucleotide that is located 500 bp upstream of the *TTP* transcription start site and TGF-β1 responsive element is critical for TGF-β1-induced epigenetic regulation of *TTP*. Thus, methylation in this promoter region inhibits TGF-β1-mediated *TTP* induction that inhibits the antiproliferative effect of TGF-β1. Researchers suggest that the methyl group added to this single CpG site within the *TTP* promoter can recruit repressor factors that promote DNA–protein interactions and can inactivate *TTP*. Transcriptional repressors, such as MeCP2, may also recruit HDAC complexes to help in repressing *TTP* expression [126]. 

Zhao and co-authors showed that MeCP2 levels are high in human HCC tissue. They found that MeCP2 inhibition by siRNA can reduce the proliferation of HepG2 cells. They also showed that MeCP2 promotes cell proliferation by activating the ERK1/2 signaling pathway while inhibiting p38 activity [127]. Wu and colleagues found that MeCP2 is overexpressed in the SMMC-7721 cell line as well as human HCC tissues and that in SMMC-7721 cells, MeCP2 silencing inhibits cell proliferation. They discovered that Melittin inhibited MeCP2, Shh, and GLI1, while it induced PTCH1, possibly resulting from induced demethylation of the *PTCH1* promoter. They reported that Melittin inhibits cell proliferation in SMMC7721 cells by downregulating MeCP2 via Shh signaling [128]. Wang and colleagues proposed a different mechanism for the MeCP2 role in HCC development. They discovered that MeCP2 and CREB1 increased the expression of HOXD3 in HCC cells by binding to its hypermethylated promoter. By binding to the heparin-binding epidermal growth factor (*HB-EGF*) promoter, HOXD3 induced *HB-EGF*, leading to increased HCC cells invasion and migration [129]. These researchers also reported that *miR-1324* is a negative regulator of MeCP2 in the sorafenib resistance of HCC. However, in these cells, the presence of circFOXM1, which releases MeCP2 from *miR-1324* inhibition, increased its expression level and thus causes sorafenib resistance [130]. 

### 4.6. MeCP2 and Prostate Cancer

**Figure 6:**
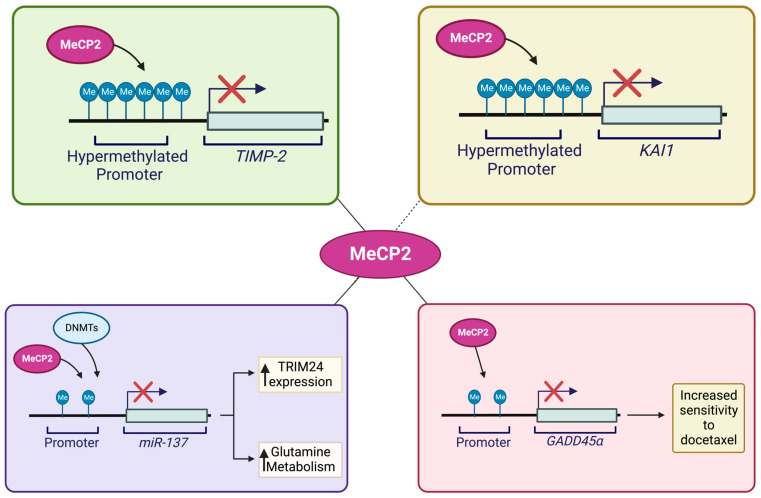
Schematic representing the suspected roles of and molecular pathways involving MeCP2 in prostate-cancer progression. The suppression of the *KAI1* metastasis suppressor gene due to alterations in methylation patterns at the *KAI1* CpG-methylation sites within the promoter region allows for the binding of MeCP2 and subsequent reduction of KAI1 in prostate cancer cells [132]. Hypermethylation of the *TIMP-2* promoter facilitates the binding of MeCP2 to affect TIMP-2 expression at the invasive and metastatic stages of prostate-cancer progression [133]. Transcriptional repression of *GADD45α* by the binding of MeCP2 to four aberrantly methylated CpG sites upstream of the proximal promoter region results in the silencing of *GADD45α*. Downregulation of MeCP2 or administration of DNMT inhibitors to increase expression of GADD45α may result in increased sensitivity to docetaxel chemotherapy [134]. Inhibition of TRIM24 may be achieved by the binding of *miR-137* to the *TRIM24* 3′-UTR promoter region. However, inhibition of *miR-137* by MeCP2 reduces *miR-137* expression via increased *miR-137* promoter methylation [135]. Illustration is generated using BioRender.com.

Prostate cancer is known as a form of cancer that may progress androgen dependently or androgen independently. Bernard and colleagues discovered that MeCP2 has a function in androgen-dependent and/or -independent proliferation in prostate cancer. This group demonstrated that raising MeCP2 expression permits androgen-dependent cells to grow androgen independently and in androgen-depleted conditions. They found that MeCP2 promotes androgen-independent proliferation in prostate cancer cells via maintaining c-Myc levels [131]. Several groups have also investigated the molecular pathways that link MeCP2 to prostate-cancer progression, as summarized in Figure 6. Specifically, *KAI1*, a metastasis suppressor gene, plays an important role in the metastasis of prostate cancer. Lee and colleagues found that changes in CpG-site methylations during the malignant progression of prostate cancer may be involved in *KAI1* reduction in prostate cancer. The researchers discovered that in prostate-cancer cells with low levels of KAI1, methyl-CpG-binding proteins including MBD2 and MeCP2 are recruited to the promoter sequences and reduce the corresponding protein levels [132]. Pulukuri and team also demonstrated that promoter hypermethylation of the *TIMP-2* gene affects its level in prostate cancer. The authors also revealed that MeCP2 binding to the promoter of TIMP-2-silenced metastatic prostate cell lines interferes with the transcriptional activity of the *TIMP-2* promoter [133]. Additionally, GADD45, which is reduced in prostate cancer, plays a key role in inhibiting the cell cycle, cell proliferation, and inducing apoptosis in cancer cells. Ramachandran and co-authors identified MeCP2 as a transcriptional repressor of GADD45 and demonstrated that decreased GADD45 was caused by atypical methylation of four sets of CpG dinucleotides located upstream of the proximal promoter. More in depth studies have found that MeCP2 binding to the methylated 5′ CpG sequences upstream of the *GADD45* promoter in Du145 cells provokes transcriptional silencing of *GADD45*. The authors also reported that increasing GADD45 expression, either through MeCP2 downregulation or treatment with DNMT inhibitors, could reduce resistance to docetaxel chemotherapy [134]. 

Bicalutamide is an effective hormone-therapy agent for prostate cancer. Nonetheless, prostate-cancer cells develop resistance to bicalutamide over time. Guan and colleagues reported the role of MeCP2 in bicalutamide resistance of prostate-cancer cells. They discovered that by binding to the 3′-UTR promoter region, *miR-137* could inhibit *TRIM24* expression, encoding for an oncogenic transcriptional activator and proliferation regulator in cancer cells. However, *miR-137* was significantly downregulated in a bicalutamide-resistant prostate-cancer cell line. Studies have shown that MeCP2 and DNMTs inhibit *miR-137* expression by increasing active methylation of the *miR-137* promoter. Meanwhile, reduced *miR-137* expression is associated with increased levels of *TRIM24* expression that causes bicalutamide-resistant expression in prostate-cancer cells [135]. 

Research studies have shown that the role of MeCP2 in cancer may go beyond the boundaries of epigenetics. Investigation into the mechanisms by which cancer cells are protected against stress implicates MeCP2 in the transactivation of putative pro-survival genes in prostate cancer. In their study, Leoh and team suggested that protein interactions between MeCP2 and lens epithelium-derived growth factor p75 (LEDGF/p75) transcriptionally activate heat-shock protein 27 (*Hsp27*) in vitro. LEDGF/p75 is considered as a stress-response protein with a protective function against cellular damage and cell death induced by oxidative stress. LEDGF/p75 is suggested to be involved in the transcriptional activation of protective genes encoding proteins including HSP27, peroxiredoxin 6 (PRDX6) αβ-crystallin, and vascular endothelial growth factor C (VEGF-C). Leoh and colleagues proposed that *Hsp27* is a target gene of both LEDGF/p75 and MeCP2. The researchers suggested that MeCP2-induced activation of the pro-survival gene *Hsp27* in prostate cancer promotes resistance to both oxidative stress-induced and chemotherapy-induced cell death, a phenomenon supporting prostatic tumorigenesis.

### 4.7. MeCP2 and Glioma

Sharma and colleagues proposed the involvement of MeCP2 in glioma by showing that MeCP2 overexpression can inhibit proliferation, migration, invasion, and adhesion while inhibiting malignant behavior in glioma cells by inhibiting pERK and BDNF and inducing GFAP [136]. In another study, Bian and co-authors reported that the MeCP2 regulatory role at the *miR-200c* promoter through interaction with SUV39H1, which causes transcriptional repression of the *miR-200c* and induces the EMT process in glioma [137].

### 4.8. MeCP2 and Oral Squamous-Cell Carcinoma

Zhang and colleagues reported that the expression level of *miR-106a*, which functions as a tumor suppressor, is reduced in the tissues and cell lines of oral squamous-cell carcinoma (OSCC). This group showed that in OSCC, *miR-106a* causes suppression of the Wnt/β-catenin signaling pathway and induces apoptosis by targeting MeCP2 [138]. 

### 4.9. MeCP2 and Renal Cell Cancer

Liu and colleagues proposed a role for MeCP2 in renal-cell cancer (RCC). They reported that the expression of MeCP2, which reduces the proliferative, migratory, and invasive abilities of RCC cells, is downregulated in RCC cell lines and tissues. Studies also showed that *miR-454*, which has a high expression level in RCC, inhibits *MECP2* transcripts by direct targeting, and, eventually, causes RCC progression [139]. 

### 4.10. MeCP2 and Lung Cancer

As mentioned earlier, the hypermethylation of the promotor regions of tumor-suppressor genes, and their subsequent downregulation through transcriptional silencing, is a common theme in tumorigenesis. In this regard, a study about aberrant 5′ CpG methylation due to overexpression of DNMTs described the role of protomer hypermethylation in the poor prognosis of lung cancer and suggests a possible link to MeCP2 [140]. Using surgically resected tissues of patients diagnosed with non-small-cell lung cancer, Lin and colleagues suggested that CpG hypermethylation of *FHIT*, *p16^INK4a^*, and *RARβ* tumor-suppressor gene promoters is associated with the overexpression of DNMT1, DNMT3A, and DNMT3B. The researchers showed transcriptional silencing of tumor-suppressor genes (*FHIT*, *p16^INK4a^*, and *RARβ*) due to direct interactions between DNMT1 and MeCP2. It was thus hypothesized that the recruitment of DNMTs to promoters occurs via DNMTs-MeCP2 complexes that function in hypermethylated regions of the tumor-suppressor genes in tumors [140].

Han and colleagues showed a link between long intergenic noncoding RNA 00518 (*LINC00518*) and MeCP2 in lung adenocarcinoma (LUAD) tumors. They showed that *LINC00518*, which is highly expressed in LUAD and is associated with poor survival, increases MeCP2 expression by inhibiting *miR-185-3p*. Such altered expressions promote cell proliferation, and subsequent tumor growth, by regulating the LUAD cell cycle [141].

## 5. Conclusions

Overall, the aforementioned research studies suggest the oncogenic properties of MeCP2 as a link between DNA methylation and different types of human cancer. Independent groups have shown that MeCP2 is increasingly presented as an emerging oncogene with involvement in several different types of human cancer. Further, these studies underscore the diversity in molecular mechanisms, in which MeCP2 could contribute to human cancer and tumorigenesis. This observed diversity may be attributed to the tissue-specific context of genes that interact with MeCP2 or in DNMTs–MeCP2 complexes. Additionally, this diversity may coincide with the major molecular pathways that are involved in each type of human cancer. Thus, the research presented by independent groups suggest that the main role of MeCP2 in cancer may be tissue- and pathway-specific. These studies offer important information towards future therapeutic strategies that are yet to be explored based on the suggested mechanisms of MeCP2-associated cancer progression.

## Abbreviations

5-aza: 5-aza-2′-deoxycytidine; ATM: ATM serine/threonine kinase; ATRX: SWI/SNF DNA helicase/ATPase; BDNF: Brain Derived Neurotrophic Factor; BMP4: Bone Morphogenetic Protein 4; BRCA1/2: Breast cancer 1/2; CD133: Prominin-like Protein 1; CHK2: Checkpoint Kinase 2; circFOXM1: Circular RNA Forkhead Box M1; CLDN6: Claudin-6; CRC: Colorectal Cancer CREB1: CAMP Responsive Element Binding Protein 1; DNMT1/3b: DNA Methyltransferase 1/3b DNMT3A: DNA Methyltransferase 3A; EMT: Epithelial-to-Mesenchymal Transition; ENPP4: Ectonucleotide Pyrophosphatase/Phosphodiesterase 4; ER: Estrogen Receptor; ERK1/2: Extracellular Signal-Regulated Protein Kinase 1/2; ESR1: Estrogen Receptor 1; FHIT: Fragile Histidine Triad Diadenosine Triphosphate; FOXF1: Forkhead box protein F1; GADD45α: Growth Arrest and DNA Damage inducible, alpha; GIT1: G-protein-coupled receptor kinase-interacting protein 1; H-RAS: Harvey Rate sarcoma virus; HB-EGF: Heparin-binding Epidermal Growth Factor; HDAC1: Histone Deacetylase-1; HDAC2: Histone Deacetylase-2; HER2: Human Epidermal Growth Factor Receptor 2; HOXD3: Homeobox D3; Hsp27: Heat Shock Protein 27; H3K9: Histone H3 lysine 9; IGF2BP2: Insulin-like Growth Factor 2 mRNA-Binding Protein 2; IL-6: Interleukin-6; KAI1: Kangai 1; KLF4: Kruppel-like Factor 4; KLK6: Human Kallikrein-related peptidase 6; LEDGF/p75: Lens Epithelium-Derived Growth Factor/p75; Linc-ROR: Long Intergenic Nonprotein Coding RNA, Regulator Of Reprogramming; LINC00518: Long Intergenic Nonprotein coding RNA 00518; LIN28A: Lin-28 homolog A; LIN28B: Lin-28 homolog B; LUAD: Lung Adenocarcinoma; m6A: N6-methyladenosine; MAPK: Mitogen Activated Protein Kinases; MBD 1/2: methyl-CpG-binding domain; MDM2: Mouse double minute 2 homolog; MeCP2: Methyl-CpG-Binding Protein 2; MEK1/2: Mitogen-activated protein kinase 1/2; METTL3: Methyltransferase 3; METTL14: Methyltransferase-like 14; *miR-22*: microRNA 22; *miR-106a*: microRNA 106a; *miR-137*: microRNA 137; *miR-185-3p*: microRNA 185-3p; *miR-194-3p*: microRNA 194-3p; *miR-200c*: microRNA 200c; *miR-212*: microRNA 212; *miR-338*: microRNA 338; *miR-338-3p*: microRNA 338-3p; *miR-338-5p*: microRNA 338-5p; *miR-454*: microRNA 454; *miR-638*: microRNA 638; *miR-1324*: microRNA 1324; MTHFD2: Methylenetetrahydrofolate Dehydrogenase (NADP+ dependent) 2; MTHFR: Methylenetetrahydrofolatereductase; MYC: MYC Proto-oncogene; MYOD1: Myogenic Differentiation 1; NOX4: NADPH Oxidase 4; OCT4: Octamer-binding Transcription factor 4; OR: Oestrogen Receptor; OSCC: Oral Squamous Cell Carcinoma; pERK: Phosphorylated Extracellular Signal-Regulated Protein Kinase; PI3K: Phosphoinositide 3-Kinase; PIP5K: Phosphatidylinositol-4-phosphate 5-kinase; PKM2: Pyruvate kinase isozymes M1/M2; PRDX6: Peroxiredoxin-6; PTEN: Phosphatase and tensin homolog; p38 MAPK: p38 Mitogen-Activated Protein Kinase; RARB2: Retinoic acid receptor beta 2; RAS: Rat sarcoma virus; RASSF1A: Ras associated domain family member 1; RB: Retinoblastoma protein; RCC: Renal Cell Cancer; RPL5: Ribosomal Protein L5; RPL11: Ribosomal Protein L11; SAM: S-Adenosyl methionine; Sin3A: SIN3 Transcription Regulator Family Member A; SIRT1: NAD-dependent deacetylase sirtuin-1; SOX2: SRY-Box Transcription Factor 2; SPI1: Spi-1 Proto-Oncogene; TGF-β1: Transforming Growth Factor β1; TIMP-2: Tissue Inhibitor of Metalloproteinase-2; TRIM24: Tripartite Motif-containing 24; TSA: Trichostatin A; TSS: Transcription Start Site; TTP: Tristetraprolin; and WNT5α/β: Wingless/Integrated 5 α/β; ZEB1: Zinc Finger E-Box Homeobox 1.

## References

[1] Delcuve G.P., Rastegar M., Davie J.R. (2009). Epigenetic control. J. Cell Physiol..

[2] Barber B.A., Rastegar M. (2010). Epigenetic control of Hox genes during neurogenesis, development, and disease. Ann. Anat..

[3] Rastegar M. (2017). Editorial (Thematic Issue: NeuroEpigenetics and Neurodevelopmental Disorders: From Molecular Mechanisms to Cell Fate Commitments of the Brain Cells and Human Disease). Curr. Top. Med. Chem..

[4] Moore L.D., Le T., Fan G. (2013). DNA Methylation and Its Basic Function. Neuropsychopharmacology.

[5] Abotaleb M., Samuel S.M., Varghese E., Varghese S., Kubatka P., Liskova A., Büsselberg D. (2018). Flavonoids in Cancer and Apoptosis. Cancers.

[6] Canadian Cancer Society (2022). Advisory in collaboration with the Canadian Cancer Society SC, Canada atPHAo. Canadian Cancer Statistics: A 2022 Special Report on Cancer Prevalence.

[7] Baxter E., Windloch K., Gannon F., Lee J.S. (2014). Epigenetic regulation in cancer progression. Cell. Biosci..

[8] Baylin S.B., Jones P.A. (2016). Epigenetic Determinants of Cancer. Cold Spring Harb. Perspect. Biol..

[9] Jiang W., Xia T., Liu C., Li J., Zhang W., Sun C. (2021). Remodeling the Epigenetic Landscape of Cancer—Application Potential of Flavonoids in the Prevention and Treatment of Cancer. Front. Oncol..

[10] Yu X., Li M., Guo C., Wu Y., Zhao L., Shi Q., Song J., Song B. (2021). Therapeutic Targeting of Cancer: Epigenetic Homeostasis. Front. Oncol..

[11] Bin Akhtar G., Buist M., Rastegar M. (2022). MeCP2 and transcriptional control of eukaryotic gene expression. Eur. J. Cell Biol..

[12] Liyanage V.R.B., Jarmasz J.S., Murugeshan N., Del Bigio M.R., Rastegar M., Davie J.R. (2014). DNA Modifications: Function and Applications in Normal and Disease States. Biology.

[13] Nishiyama A., Nakanishi M. (2021). Navigating the DNA methylation landscape of cancer. Trends Genet..

[14] Vuu Y.M., Roberts C.T., Rastegar M. (2023). MeCP2 Is an Epigenetic Factor That Links DNA Methylation with Brain Metabolism. Int. J. Mol. Sci..

[15] Mahmood N., Rabbani S.A. (2019). DNA Methylation Readers and Cancer: Mechanistic and Therapeutic Applications. Front. Oncol..

[16] McCabe M.T., Brandes J.C., Vertino P.M. (2009). Cancer DNA methylation: Molecular mechanisms and clinical implications. Clin. Cancer Res. Off. J. Am. Assoc. Cancer Res..

[17] Herman J.G., Baylin S.B. (2003). Gene silencing in cancer in association with promoter hypermethylation. N. Engl. J. Med..

[18] Cui D., Xu X. (2018). DNA Methyltransferases, DNA Methylation, and Age-Associated Cognitive Function. Int. J. Mol. Sci..

[19] Hervouet E., Peixoto P., Delage-Mourroux R., Boyer-Guittaut M., Cartron P.-F. (2018). Specific or not specific recruitment of DNMTs for DNA methylation, an epigenetic dilemma. Clin. Epigenetics.

[20] Nowacka-Zawisza M., Wiśnik E. (2017). DNA methylation and histone modifications as epigenetic regulation in prostate cancer. Oncol. Rep..

[21] Subramaniam D., Thombre R., Dhar A., Anant S. (2014). DNA Methyltransferases: A Novel Target for Prevention and Therapy. Front. Oncol..

[22] Bird A.P. (1986). CpG-rich islands and the function of DNA methylation. Nature.

[23] Santini V., Kantarjian H.M., Issa J.P. (2001). Changes in DNA methylation in neoplasia: Pathophysiology and therapeutic implications. Ann. Intern. Med..

[24] Ellis J., Hotta A., Rastegar M. (2007). Retrovirus silencing by an epigenetic TRIM. Cell.

[25] Zachariah R.M., Rastegar M. (2012). Linking epigenetics to human disease and Rett syndrome: The emerging novel and challenging concepts in MeCP2 research. Neural Plast..

[26] Chahrour M., Jung S.Y., Shaw C., Zhou X., Wong S.T., Qin J., Zoghbi H.Y. (2008). MeCP2, a key contributor to neurological disease, activates and represses transcription. Science.

[27] Liyanage V.R., Zachariah R.M., Davie J.R., Rastegar M. (2015). Ethanol deregulates Mecp2/MeCP2 in differentiating neural stem cells via interplay between 5-methylcytosine and 5-hydroxymethylcytosine at the Mecp2 regulatory elements. Exp. Neurol..

[28] Liyanage V.R.B., Olson C.O., Zachariah R.M., Davie J.R., Rastegar M. (2019). DNA Methylation Contributes to the Differential Expression Levels of Mecp2 in Male Mice Neurons and Astrocytes. Int. J. Mol. Sci..

[29] Olson C.O., Zachariah R.M., Ezeonwuka C.D., Liyanage V.R., Rastegar M. (2014). Brain region-specific expression of MeCP2 isoforms correlates with DNA methylation within Mecp2 regulatory elements. PLoS ONE..

[30] Xu W., Liyanage V.R.B., MacAulay A., Levy R.D., Curtis K., Olson C.O., Zachariah R.M., Amiri S., Buist M., Hicks G.G. (2019). Genome-Wide Transcriptome Landscape of Embryonic Brain-Derived Neural Stem Cells Exposed to Alcohol with Strain-Specific Cross-Examination in BL6 and CD1 Mice. Sci. Rep..

[31] Liyanage V.R., Zachariah R.M., Rastegar M. (2013). Decitabine alters the expression of Mecp2 isoforms via dynamic DNA methylation at the Mecp2 regulatory elements in neural stem cells. Mol. Autism..

[32] Buist M., El Tobgy N., Shevkoplyas D., Genung M., Sher A.A., Pejhan S., Rastegar M. (2022). Differential Sensitivity of the Protein Translation Initiation Machinery and mTOR Signaling to MECP2 Gain- and Loss-of-Function Involves MeCP2 Isoform-Specific Homeostasis in the Brain. Cells.

[33] Olson C.O., Pejhan S., Kroft D., Sheikholeslami K., Fuss D., Buist M., Ali Sher A., Del Bigio M.R., Sztainberg Y., Siu V.M. (2018). MECP2 Mutation Interrupts Nucleolin-mTOR-P70S6K Signaling in Rett Syndrome Patients. Front. Genet..

[34] Li Y., Wang H., Muffat J., Cheng A.W., Orlando D.A., Loven J., Kwok S.M., Feldman D.A., Bateup H.S., Gao Q. (2013). Global transcriptional and translational repression in human-embryonic-stem-cell-derived Rett syndrome neurons. Cell Stem Cell.

[35] Tong D., Zhang J., Wang X., Li Q., Liu L.Y., Yang J., Guo B., Ni L., Zhao L. (2020). MeCP2 facilitates breast cancer growth via promoting ubiquitination-mediated P53 degradation by inhibiting RPL5/RPL11 transcription. Oncogenesis.

[36] Pandey S., Pruitt K. (2017). Functional assessment of MeCP2 in Rett syndrome and cancers of breast, colon, and prostate. Biochem. Cell Biol..

[37] Razin A., Shemer R. (1995). DNA methylation in early development. Hum. Mol. Genet..

[38] Olynik B.M., Rastegar M. (2012). The genetic and epigenetic journey of embryonic stem cells into mature neural cells. Front. Genet..

[39] Rastegar M., Yasui D.H. (2021). Editorial: Epigenetic Mechanisms and Their Involvement in Rare Diseases. Front. Genet..

[40] Liyanage V.R., Curtis K., Zachariah R.M., Chudley A.E., Rastegar M. (2017). Overview of the Genetic Basis and Epigenetic Mechanisms that Contribute to FASD Pathobiology. Curr. Top. Med. Chem..

[41] Bestor T.H., Verdine G.L. (1994). DNA methyltransferases. Curr. Opin. Cell Biol..

[42] Feinberg A.P., Vogelstein B. (1983). Hypomethylation distinguishes genes of some human cancers from their normal counterparts. Nature.

[43] Sakai T., Toguchida J., Ohtani N., Yandell D.W., Rapaport J.M., Dryja T.P. (1991). Allele-specific hypermethylation of the retinoblastoma tumor-suppressor gene. Am. J. Hum. Genet..

[44] Wajed S.A., Laird P.W., DeMeester T.R. (2001). DNA methylation: An alternative pathway to cancer. Ann. Surg..

[45] Hentze J.L., Høgdall C.K., Høgdall E.V. (2019). Methylation and ovarian cancer: Can DNA methylation be of diagnostic use?. Mol. Clin. Oncol..

[46] Rodriguez J., Frigola J., Vendrell E., Risques R.A., Fraga M.F., Morales C., Moreno V., Esteller M., Capellà G., Ribas M. (2006). Chromosomal instability correlates with genome-wide DNA demethylation in human primary colorectal cancers. Cancer Res..

[47] Lewis J.D., Meehan R.R., Henzel W.J., Maurer-Fogy I., Jeppesen P., Klein F., Bird A. (1992). Purification, sequence, and cellular localization of a novel chromosomal protein that binds to methylated DNA. Cell.

[48] Meehan R.R., Lewis J.D., Bird A.P. (1992). Characterization of MeCP2, a vertebrate DNA binding protein with affinity for methylated DNA. Nucleic Acids Res..

[49] Della Ragione F., Filosa S., Scalabrì F., D’Esposito M. (2012). MeCP2 as a genome-wide modulator: The renewal of an old story. Front. Genet..

[50] Good K.V., Vincent J.B., Ausió J. (2021). MeCP2: The Genetic Driver of Rett Syndrome Epigenetics. Front. Genet..

[51] Bianciardi L., Fichera M., Failla P., Di Marco C., Grozeva D., Mencarelli M.A., Spiga O., Mari F., Meloni I., Raymond L. (2016). MECP2 missense mutations outside the canonical MBD and TRD domains in males with intellectual disability. J. Hum. Genet..

[52] Shevkoplyas D., Vuu Y.M., Davie J.R., Rastegar M. (2022). The Chromatin Structure at the MECP2 Gene and In Silico Prediction of Potential Coding and Non-Coding MECP2 Splice Variants. Int. J. Mol. Sci..

[53] Kriaucionis S., Bird A. (2004). The major form of MeCP2 has a novel N-terminus generated by alternative splicing. Nucleic Acids Res..

[54] Pejhan S., Del Bigio M.R., Rastegar M. (2020). The MeCP2E1/E2-BDNF-miR132 Homeostasis Regulatory Network Is Region-Dependent in the Human Brain and Is Impaired in Rett Syndrome Patients. Front. Cell Dev. Biol..

[55] Buist M., Fuss D., Rastegar M. (2021). Transcriptional Regulation of MECP2E1-E2 Isoforms and BDNF by Metformin and Simvastatin through Analyzing Nascent RNA Synthesis in a Human Brain Cell Line. Biomolecules..

[56] Sheikholeslami K., Ali Sher A., Lockman S., Kroft D., Ganjibakhsh M., Nejati-Koshki K., Shojaei S., Ghavami S., Rastegar M. (2019). Simvastatin Induces Apoptosis in Medulloblastoma Brain Tumor Cells via Mevalonate Cascade Prenylation Substrates. Cancers.

[57] Dastidar S.G., Bardai F.H., Ma C., Price V., Rawat V., Verma P., Narayanan V., D’Mello S.R. (2012). Isoform-specific toxicity of Mecp2 in postmitotic neurons: Suppression of neurotoxicity by FoxG1. J. Neurosci..

[58] Lipsick J. (2020). A History of Cancer Research: Tumor Suppressor Genes. Cold Spring Harb. Perspect. Biol..

[59] Osborne C., Wilson P., Tripathy D. (2004). Oncogenes and tumor suppressor genes in breast cancer: Potential diagnostic and therapeutic applications. Oncologist.

[60] Arizmendi-Izazaga A., Martinez-Baltazar R., Liborio-Bautista A., Olea-Flores M., Ortiz-Ortiz J., Navarro-Tito N. (2023). The NRSF/REST transcription factor in hallmarks of cancer: From molecular mechanisms to clinical relevance. Biochimie.

[61] Talia K.L., Banet N., Buza N. (2023). The role of HER2 as a therapeutic biomarker in gynaecological malignancy: Potential for use beyond uterine serous carcinoma. Pathology.

[62] Jang H., Seo A.N., Kim M. (2022). Clinicopathological Characteristics of Advanced Epstein-Barr Virus-associated Gastric Cancer Highlighting Aberrant p53 Expression. Anticancer. Res..

[63] Lhermitte B., Blandin A.F., Coca A., Guerin E., Durand A., Entz-Werle N. (2021). Signaling pathway deregulation and molecular alterations across pediatric medulloblastomas. Neurochirurgie.

[64] Vriend J., Rastegar M. (2020). Ubiquitin ligases and medulloblastoma: Genetic markers of the four consensus subgroups identified through transcriptome datasets. Biochim. Biophys. Acta Mol. Basis Dis..

[65] Boveri T. (1914). Zur Frage der Entstehung Maligner Tumoren.

[66] Nowell P.C. (1962). The minute chromosome (Phl) in chronic granulocytic leukemia. Blut.

[67] Knudson A.G. (1971). Mutation and cancer: Statistical study of retinoblastoma. Proc. Natl. Acad. Sci. USA..

[68] Cavenee W.K., Dryja T.P., Phillips R.A., Benedict W.F., Godbout R., Gallie B.L., Murphree A.L., Strong L.C., White R.L. (1983). Expression of recessive alleles by chromosomal mechanisms in retinoblastoma. Nature.

[69] Friend S.H., Bernards R., Rogelj S., Weinberg R.A., Rapaport J.M., Albert D.M., Dryja T.P. (1986). A human DNA segment with properties of the gene that predisposes to retinoblastoma and osteosarcoma. Nature.

[70] Stehelin D., Varmus H.E., Bishop J.M. (1976). Vogt PKDNA related to the transforming gene(s) of avian sarcoma viruses is present in normal avian, D.N.A. Nature.

[71] Reddy E.P., Reynolds R.K., Santos E., Barbacid M. (1982). A point mutation is responsible for the acquisition of transforming properties by the T24 human bladder carcinoma oncogene. Nature.

[72] Bell D.W. (2010). Our changing view of the genomic landscape of cancer. J. Pathol..

[73] Wu C.H., Gordon J., Rastegar M., Ogretmen B., Safa A.R. (2002). Proteinase-3, a serine protease which mediates doxorubicin-induced apoptosis in the HL-60 leukemia cell line, is downregulated in its doxorubicin-resistant variant. Oncogene.

[74] Wu C.H., Rastegar M., Gordon J., Safa A.R. (2001). beta(2)-microglobulin induces apoptosis in HL-60 human leukemia cell line and its multidrug resistant variants overexpressing MRP1 but lacking Bax or overexpressing P-glycoprotein. Oncogene.

[75] Gordon J., Wu C.H., Rastegar M., Safa A.R. (2003). Beta2-microglobulin induces caspase-dependent apoptosis in the CCRF-HSB-2 human leukemia cell line independently of the caspase-3, -8 and -9 pathways but through increased reactive oxygen species. Int. J. Cancer.

[76] Karpel H., Slomovitz B., Coleman R.L., Pothuri B. (2023). Biomarker-driven therapy in endometrial cancer. Int. J. Gynecol. Cancer.

[77] Sherr C.J. (2004). Principles of tumor suppression. Cell.

[78] Loboda A.P., Adonin L.S., Zvereva S.D., Guschin D.Y., Korneenko T.V., Telegina A.V., Kondratieva O.K., Frolova S.E., Pestov N.B., Barlev N.A. (2023). BRCA Mutations-The Achilles Heel of Breast, Ovarian and Other Epithelial Cancers. Int. J. Mol. Sci..

[79] Steffen C.L., Kaya P., Schaffner-Reckinger E., Abankwa D. (2023). Eliminating oncogenic RAS: Back to the future at the drawing board. Biochem. Soc. Trans..

[80] Nambiar M., Kari V., Raghavan S.C. (2008). Chromosomal translocations in cancer. Biochim. Biophys. Acta.

[81] Storlazzi C.T., Lonoce A., Guastadisegni M.C., Trombetta D., D’Addabbo P., Daniele G., L’Abbate A., Macchia G., Surace C., Kok K. (2010). Gene amplification as double minutes or homogeneously staining regions in solid tumors: Origin and structure. Genome Res..

[82] Freudenberg J.A., Wang Q., Katsumata M., Drebin J., Nagatomo I., Greene M.I. (2009). The role of HER2 in early breast cancer metastasis and the origins of resistance to HER2-targeted therapies. Exp. Mol. Pathol..

[83] Bornkamm G.W., Polack A., Eick D., Berger R., Lenoir G.M. (1987). [Chromosome translocations and Epstein-Barr virus in Burkitt’s lymphoma]. Onkologie..

[84] Howell G.M., Hodak S.P., Yip L. (2013). RAS mutations in thyroid cancer. Oncologist.

[85] Pejhan S., Rastegar M. (2021). Role of DNA Methyl-CpG-Binding Protein MeCP2 in Rett Syndrome Pathobiology and Mechanism of Disease. Biomolecules.

[86] Rastegar M., Hotta A., Pasceri P., Makarem M., Cheung A.Y., Elliott S., Park K.J., Adachi M., Jones F.S., Clarke I.D. (2009). MECP2 isoform-specific vectors with regulated expression for Rett syndrome gene therapy. PLoS ONE.

[87] Amir R.E., Van den Veyver I.B., Wan M., Tran C.Q., Francke U., Zoghbi H.Y. (1999). Rett syndrome is caused by mutations in X-linked MECP2, encoding methyl-CpG-binding protein 2. Nat. Genet..

[88] Ezeonwuka C.D., Rastegar M. (2014). MeCP2-Related Diseases and Animal Models. Diseases.

[89] Yasui D.H., Gonzales M.L., Aflatooni J.O., Crary F.K., Hu D.J., Gavino B.J., Golub M.S., Vincent J.B., Carolyn Schanen N., Olson C.O. (2014). Mice with an isoform-ablating Mecp2 exon 1 mutation recapitulate the neurologic deficits of Rett syndrome. Hum. Mol. Genet..

[90] Neupane M., Clark A.P., Landini S., Birkbak N.J., Eklund A.C., Lim E., Culhane A.C., Barry W.T., Schumacher S.E., Beroukhim R. (2016). MECP2 Is a Frequently Amplified Oncogene with a Novel Epigenetic Mechanism That Mimics the Role of Activated RAS in Malignancy. Cancer Discov..

[91] Luo D., Ge W. (2020). MeCP2 Promotes Colorectal Cancer Metastasis by Modulating ZEB1 Transcription. Cancers.

[92] Zhang J., Zhao J., Gao N., Wang Y., Chen Y., Han J. (2017). MECP2 expression in gastric cancer and its correlation with clinical pathological parameters. Medicine.

[93] Zhao L., Liu Y., Tong D., Qin Y., Yang J., Xue M., Du N., Liu L., Guo B., Hou N. (2017). MeCP2 Promotes Gastric Cancer Progression Through Regulating FOXF1/Wnt5a/β-Catenin and MYOD1/Caspase-3 Signaling Pathways. EBioMedicine.

[94] Fang J.Y., Cheng Z.H., Chen Y.X., Lu R., Yang L., Zhu H.Y., Lu L.G. (2004). Expression of Dnmt1, demethylase, MeCP2 and methylation of tumor-related genes in human gastric cancer. World J. Gastroenterol..

[95] Patra S.K., Patra A., Zhao H., Carroll P., Dahiya R. (2003). Methyl-CpG-DNA binding proteins in human prostate cancer: Expression of CXXC sequence containing MBD1 and repression of MBD2 and MeCP2. Biochem. Biophys. Res. Commun..

[96] Müller H.M., Fiegl H., Goebel G., Hubalek M.M., Widschwendter A., Müller-Holzner E., Marth C., Widschwendter M. (2003). MeCP2 and MBD2 expression in human neoplastic and non-neoplastic breast tissue and its association with oestrogen receptor status. Br. J. Cancer.

[97] Song N., Li K., Wang Y., Chen Z., Shi L. (2016). Lentivirus-mediated knockdown of MeCP2 inhibits the growth of colorectal cancer cells in vitro. Mol. Med. Rep..

[98] Lin H.-Y., Wu H.-J., Chen S.-Y., Hou M.-F., Lin C.-S., Chu P.-Y. (2022). Epigenetic therapy combination of UNC0638 and CI-994 suppresses breast cancer via epigenetic remodeling of BIRC5 and GADD45A. Biomed. Pharmacother..

[99] Jiang Y., Jiang Z., Wang M., Ma L. (2022). Current understandings and clinical translation of nanomedicines for breast cancer therapy. Adv. Drug Deliv. Rev..

[100] Castro-Piedras I., Vartak D., Sharma M., Pandey S., Casas L., Molehin D., Rasha F., Fokar M., Nichols J., Almodovar S. (2020). Identification of Novel MeCP2 Cancer-Associated Target Genes and Post-Translational Modifications. Front. Oncol..

[101] Billard L.-M., Magdinier F., Lenoir G.M., Frappart L., Dante R. (2002). MeCP2 and MBD2 expression during normal and pathological growth of the human mammary gland. Oncogene.

[102] Rasti M., Arabsolghar R., Khatooni Z., Mostafavi-Pour Z. (2012). p53 Binds to estrogen receptor 1 promoter in human breast cancer cells. Pathol. Oncol. Res..

[103] Sharma D., Blum J., Yang X., Beaulieu N., Macleod A.R., Davidson N.E. (2005). Release of methyl CpG binding proteins and histone deacetylase 1 from the estrogen receptor α (ER) promoter upon reactivation in ER-negative human breast cancer cells. Mol. Endocrinol..

[104] Jiang W., Liang Y.-L., Liu Y., Chen Y.-Y., Yang S.-T., Li B.-R., Yu Y.-X., Lyu Y., Wang R. (2020). MeCP2 inhibits proliferation and migration of breast cancer via suppression of epithelial-mesenchymal transition. J. Cell Mol. Med..

[105] Pampalakis G., Prosnikli E., Agalioti T., Vlahou A., Zoumpourlis V., Sotiropoulou G. (2009). A Tumor-Protective Role for Human Kallikrein-Related Peptidase 6 in Breast Cancer Mediated by Inhibition of Epithelial-to-Mesenchymal Transition. Cancer Res..

[106] Pandey S., Simmons G.E., Jr Malyarchuk S., Calhoun T.N., Pruitt K. (2015). A novel MeCP2 acetylation site regulates interaction with ATRX and HDAC1. Genes Cancer.

[107] Liu Y., Jin X., Li Y., Ruan Y., Lu Y., Yang M., Lin D., Song P., Guo Y., Zhao S. (2016). DNA methylation of claudin-6 promotes breast cancer cell migration and invasion by recruiting MeCP2 and deacetylating H3Ac and H4Ac. J. Exp. Clin. Cancer Res..

[108] Zhou Q., Guo J., Huang W., Yu X., Xu C., Long X. (2020). Linc-ROR promotes the progression of breast cancer and decreases the sensitivity to rapamycin through miR-194-3p targeting MECP2. Mol Oncol..

[109] Ballestar E., Paz M.F., Valle L., Wei S., Fraga M.F., Espada J., Cigudosa J.C., Huang T.H.-M., Esteller M. (2003). Methyl-CpG binding proteins identify novel sites of epigenetic inactivation in human cancer. EMBO J..

[110] Yilmaz T.U., Güneş A., Pösteki G., Okay E. (2014). Rett syndrome with colon cancer presented with sigmoid volvulus: Report of a case. Int. J. Surg. Case Rep..

[111] Wang S., Gan M., Chen C., Zhang Y., Kong J., Zhang H., Lai M. (2021). Methyl CpG binding protein 2 promotes colorectal cancer metastasis by regulating N(6)-methyladenosine methylation through methyltransferase-like 14. Cancer Sci..

[112] Darwanto A., Kitazawa R., Maeda S., Kitazawa S. (2003). MeCP2 and promoter methylation cooperatively regulate E-cadherin gene expression in colorectal carcinoma. Cancer Sci..

[113] Chen T., Cai S.-L., Li J., Qi Z.-P., Li X.-Q., Ye L.-C., Xie X.-F., Hou Y.-Y., Yao L.-Q., Xu M.-D. (2017). Mecp2-mediated Epigenetic Silencing of miR-137 Contributes to Colorectal Adenoma-Carcinoma Sequence and Tumor Progression via Relieving the Suppression of c-Met. Sci. Rep..

[114] Xie Y., Gao J. (2021). Expression and clinical significance of methyl-CpG-binding protein 2 in pancreas cancer and surrounding tissue. Chin. J. Pancreatol..

[115] Wang H., Li J., He J., Liu Y., Feng W., Zhou H., Zhou M., Wei H., Lu Y., Peng W. (2020). Methyl-CpG-binding protein 2 drives the Furin/TGF-β1/Smad axis to promote epithelial–mesenchymal transition in pancreatic cancer cells. Oncogenesis.

[116] Xu M., Bian S., Li J., He J., Chen H., Ge L., Jiao Z., Zhang Y., Peng W., Du F. (2016). MeCP2 suppresses LIN28A expression via binding to its methylated-CpG islands in pancreatic cancer cells. Oncotarget.

[117] Dandrea M., Donadelli M., Costanzo C., Scarpa A., Palmieri M. (2009). MeCP2/H3meK9 are involved in IL-6 gene silencing in pancreatic adenocarcinoma cell lines. Nucleic Acids Res..

[118] Zhao L., Wang X., Yang J., Jiang Q., Zhang J., Qin Y., Wang L., Liu L., Ni L., Tong D. MECP2 Promotes Migration and Invasion of Gastric Cancer Cells via Modulating the Notch1/c-Myc/mTOR Signaling Pathways by Suppressing FBXW7 Transcription. 2022. https://assets.researchsquare.com/files/rs-48700/v1/ca06d91a-1674-4ad5-9c79-3a5ba919317a.pdf?c=1631847938.

[119] Zhang X.Y., Xu Y.Y., Chen W.Y. (2020). MicroRNA-1324 inhibits cell proliferative ability and invasiveness by targeting MECP2 in gastric cancer. Eur. Rev. Med. Pharmacol. Sci..

[120] Zhao L.Y., Tong D.D., Xue M., Ma H.L., Liu S.Y., Yang J., Liu Y.X., Guo B., Ni L., Liu L.Y. (2017). MeCP2, a target of miR-638, facilitates gastric cancer cell proliferation through activation of the MEK1/2-ERK1/2 signaling pathway by upregulating GIT1. Oncogenesis.

[121] Wada R., Akiyama Y., Hashimoto Y., Fukamachi H., Yuasa Y. (2010). miR-212 is downregulated and suppresses methyl-CpG-binding protein MeCP2 in human gastric cancer. Int. J. Cancer.

[122] Zhu F., Wu Q., Ni Z., Lei C., Li T., Shi Y. (2018). miR-19a/b and MeCP2 repress reciprocally to regulate multidrug resistance in gastric cancer cells. Int. J. Mol. Med..

[123] Qin Y., Ma X., Guo C., Cai S., Ma H., Zhao L. (2022). MeCP2 confers 5-fluorouracil resistance in gastric cancer via upregulating the NOX4/PKM2 pathway. Cancer Cell Int..

[124] Tong D., Zhang J., Wang X., Li Q., Liu L., Lu A., Guo B., Yang J., Ni L., Qin H. (2020). MiR-22, regulated by MeCP2, suppresses gastric cancer cell proliferation by inducing a deficiency in endogenous S-adenosylmethionine. Oncogenesis.

[125] Tong D., Zhao L., He K., Sun H., Cai D., Ni L., Sun R., Chang Se Song T., Huang C. (2016). MECP2 promotes the growth of gastric cancer cells by suppressing miR-338-mediated antiproliferative effect. Oncotarget.

[126] Sohn B.H., Park I.Y., Lee J.J., Yang S.J., Jang Y.J., Park K.C., Kim D.J., Lee D.C., Sohn H.A., Kim T.W. (2010). Functional switching of TGF-beta1 signaling in liver cancer via epigenetic modulation of a single CpG site in TTP promoter. Gastroenterology.

[127] Zhao L.Y., Zhang J., Guo B., Yang J., Han J., Zhao X.G., Wang X.F., Liu L.Y., Li Z.F., Song T.S. (2013). MECP2 promotes cell proliferation by activating ERK1/2 and inhibiting p38 activity in human hepatocellular carcinoma HEPG2 cells. Cell. Mol. Biol..

[128] Wu X., Zhao B., Cheng Y., Yang Y., Huang C., Meng X., Wu B., Zhang L., Lv X., Li J. (2015). Melittin induces PTCH1 expression by down-regulating MeCP2 in human hepatocellular carcinoma SMMC-7721 cells. Toxicol. Appl. Pharmacol..

[129] Wang L., Gao Y., Tong D., Wang X., Guo C., Guo B., Yang Y., Zhao L., Zhang J., Yang J. (2021). MeCP2 drives hepatocellular carcinoma progression via enforcing HOXD3 promoter methylation and expression through the HB-EGF/EGFR pathway. Mol. Oncol..

[130] Weng H., Zeng L., Cao L., Chen T., Li Y., Xu Y., Peng Y., Ye Y. (2021). circFOXM1 contributes to sorafenib resistance of hepatocellular carcinoma cells by regulating MECP2 via miR-1324. Mol. Ther. Nucleic Acids.

[131] Bernard D., Gil J., Dumont P., Rizzo S., Monté D., Quatannens B., Hudson D., Visakorpi T., Fuks F., de Launoit Y. (2006). The methyl-CpG-binding protein MECP2 is required for prostate cancer cell growth. Oncogene.

[132] Lee J., Lee M.S., Jeoung D.I., Kim Y.M., Lee H. (2017). Promoter CpG-Site Methylation of the KAI1 Metastasis Suppressor Gene Contributes to Its Epigenetic Repression in Prostate Cancer. Prostate.

[133] Pulukuri S.M., Patibandla S., Patel J., Estes N., Rao J.S. (2007). Epigenetic inactivation of the tissue inhibitor of metalloproteinase-2 (TIMP-2) gene in human prostate tumors. Oncogene.

[134] Ramachandran K., Gopisetty G., Gordian E., Navarro L., Hader C., Reis I.M., Schulz W.A., Singal R. (2009). Methylation-Mediated Repression of GADD45α in Prostate Cancer and Its Role as a Potential Therapeutic Target. Cancer Res..

[135] Guan Y., Guan X., An H., Baihetiya A., Wang W., Shao W., Yang H., Wang Y. (2019). Epigenetic silencing of miR-137 induces resistance to bicalutamide by targeting TRIM24 in prostate cancer cells. Am. J. Transl. Res..

[136] Sharma K., Singh J., Frost E.E., Pillai P.P. (2018). MeCP2 overexpression inhibits proliferation, migration and invasion of C6 glioma by modulating ERK signaling and gene expression. Neurosci. Lett..

[137] Bian E., Chen X., Xu Y., Ji X., Cheng M., Wang H., Fang Z., Zhao B. (2019). A central role for MeCP2 in the epigenetic repression of miR-200c during epithelial-to-mesenchymal transition of glioma. J. Exp. Clin. Cancer Res..

[138] Zhang N., Wei Z.-L., Yin J., Zhang L., Wang J., Jin Z.-L. (2018). MiR-106a* inhibits oral squamous cell carcinoma progression by directly targeting MeCP2 and suppressing the Wnt/β-Catenin signaling pathway. Am. J. Transl. Res..

[139] Liu H., Liu Q.-L., Zhai T.-S., Lu J., Dong Y.-Z., Xu Y.-F. (2020). Silencing miR-454 suppresses cell proliferation, migration and invasion via directly targeting MECP2 in renal cell carcinoma. Am. J. Transl. Res..

[140] Lin R.K., Hsu H.S., Chang J.W., Chen C.Y., Chen J.T., Wang Y.C. (2007). Alteration of DNA methyltransferases contributes to 5′CpG methylation and poor prognosis in lung cancer. Lung Cancer.

[141] Han X., Wu J., Zhang Y., Song J., Shi Z., Chang H. (2021). LINC00518 Promotes Cell Proliferation by Regulating the Cell Cycle of Lung Adenocarcinoma Through miR-185-3p Targeting MECP2. Front. Oncol..

